# MiR-29b may suppresses peritoneal metastases through inhibition of the mesothelial–mesenchymal transition (MMT) of human peritoneal mesothelial cells

**DOI:** 10.1038/s41598-021-04065-2

**Published:** 2022-01-07

**Authors:** Yuki Kimura, Hideyuki Ohzawa, Hideyo Miyato, Yuki Kaneko, Akira Saito, Kazuya Takahashi, Mineyuki Tojo, Hironori Yamaguchi, Kentaro Kurashina, Shin Saito, Yoshinori Hosoya, Alan Kawarai Lefor, Naohiro Sata, Joji Kitayama

**Affiliations:** 1 Department of Surgery, Jichi Medical University, Shimotsuke, Tochigi, Japan; 2 Department of Clinical Oncology, Jichi Medical University, Shimotsuke, Tochigi, Japan; 3 Center for Clinical Research, Jichi Medical University Hospital, Shimotsuke, Tochigi, Japan

**Keywords:** Cancer, Cell biology, Gastroenterology

## Abstract

Peritoneal dissemination is a major metastatic pathway for gastrointestinal and ovarian malignancies. The miR-29b family is downregulated in peritoneal fluids in patients with peritoneal metastases (PM). We examined the effect of miR-29b on mesothelial cells (MC) which play critical a role in the development of PM through mesothelial-mesenchymal transition (MMT). Human peritoneal mesothelial cells (HPMCs) were isolated from surgically resected omental tissue and MMT induced by stimulation with 10 ng/ml TGF-β1. MiR-29b mimics and negative control miR were transfected by lipofection using RNAiMAX and the effects on the MMT evaluated in vitro*.* HPMC produced substantial amounts of miR-29b which was markedly inhibited by TGF-β1. TGF-β1 stimulation of HPMC induced morphological changes with decreased expression of E-cadherin and calretinin, and increased expression of vimentin and fibronectin. TGF-β1 also enhanced proliferation and migration of HPMC as well as adhesion of tumor cells in a fibronectin dependent manner. However, all events were strongly abrogated by simultaneous transfection of miR-29b. MiR-29b inhibits TGF-β1 induced MMT and replacement of miR-29b in the peritoneal cavity might be effective to prevent development of PM partly through the effects on MC.

## Introduction

The peritoneum is a well-known metastatic site for gastrointestinal and ovarian malignancies^1,2^. Peritoneal carcinomatosis often develops which is associated with an extremely poor prognosis^3–5^. Although, peritoneal metastases (PM) are likely to develop from free intraperitoneal tumor cells exfoliated from the serosal surface of a primary tumor^6,7^, the mechanisms underlying the process leading to the development of PM have not been fully elucidated.

The peritoneum is composed of a single layer of flat mesothelial cells (MC) and a thin layer of sub-mesothelial connective tissue. An intact MC layer is believed to act as the first barrier against bacterial invasion and tumor attachment. However, after mechanical damage or peritoneal dialysis, MC lose their intercellular junctions, and increase their migratory capacity, producing a large amount of extracellular matrix components and a wide range of inflammatory, profibrotic and angiogenic factors, causing post-surgical peritoneal adhesions^8^ or peritoneal fibrosis^9,10^. This phenomenon, named as mesothelial–mesenchymal transition (MMT), has been shown to be a critical step in the process of the development of PM^11–14^.

MicroRNAs (miRNAs) are a class of small noncoding RNA molecules, 20–23 nucleotides in length, that regulate post-transcription gene expression by interfering with the translation of multiple target mRNAs and play important roles in the regulation of a variety of cell functions. In the miR-29 family, their function and biological significance have been extensively analyzed^15–17^. In oncology, miR-29s have been shown to induce cell cycle arrest and apoptosis of tumor cells^18^, suppress epithelial mesenchymal transition (EMT)^19,20^, and inhibit angiogenesis^19,21,22^, suggesting that miR-29s predominantly function as tumor suppressor. In fact, miR-29 is reported to be downregulated in various tumors as compared with normal counterparts^23^. We consistently found that the miR-29 family in exosomes derived from peritoneal fluid was markedly downregulated in patients with peritoneal metastases from gastric cancer^24^. However, the role of miR-29s in the development of PM is not fully understood. In this study, we evaluated the effects of miR-29b on the cellular function of MC to examine its possible roles in the development of PM.

## Results

### Expression of miR-29b in HPMC was decreased by TGF-β1

**Figure 1:**
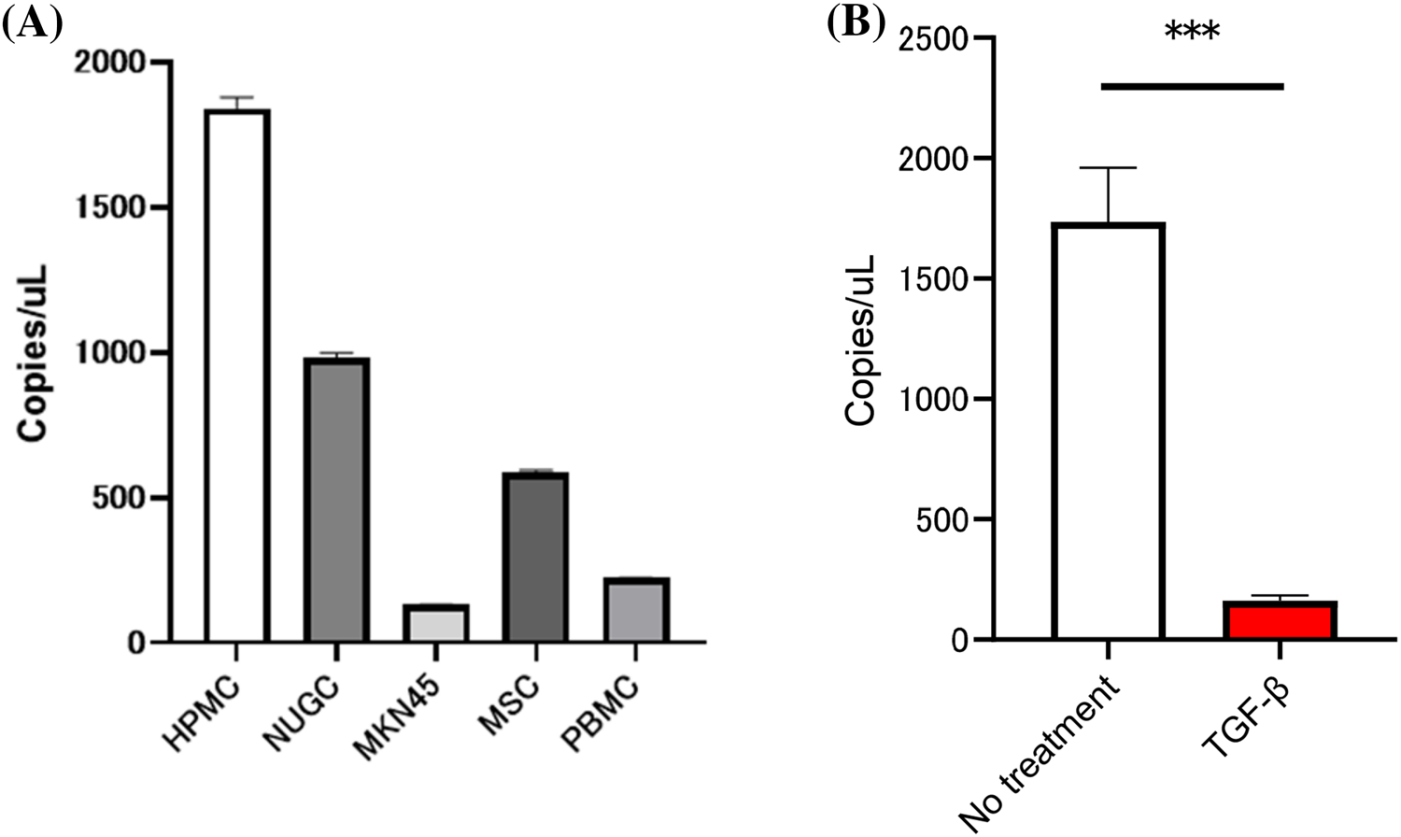
(**A**) Expression of miR-29b in cell lines and human peritoneal mesothelial cells (HPMC). Total miR-29b was quantified using the digital droplet polymerase chain reaction (dd-PCR) system. B. HPMC were cultured in the presence of 10 ng/ml TGF-β1 or without was quantified with dd-PCR. Data show mean ± standard deviation in 3 different experiments. ***p < 0.001.

HPMC were isolated from omental tissue obtained from patients who underwent sleeve gastrectomy and cultured in DMEM supplemented with 15% FBS. Digital PCR analysis revealed that HPMC produced substantial levels of miR-29b, which were more than those from gastric cancer cells, MKN45 and NUGC as well as peripheral blood mononuclear cells (PBMC) and mesenchymal stem cells (MSC) (Fig. 1A). However, when HPMC were cultured with 10 ng/ml TGF-β1 for 24 h, the expression of miR29b was markedly decreased (Fig. 1B).

### Transfection of miR-29b mimic suppresses phenotypic changes of HPMC by TGF-β1

The HPMC were transfected with miR-29b mimic or negative control miR (NC) using lipofectamine RNAiMAX. We found 50 nM miRs are enough for the transfection to more than 90% of the HPMC and expression levels of miR-29b was significantly upregulated with digital PCR system (Supplementary Fig. 1).

**Figure 2:**
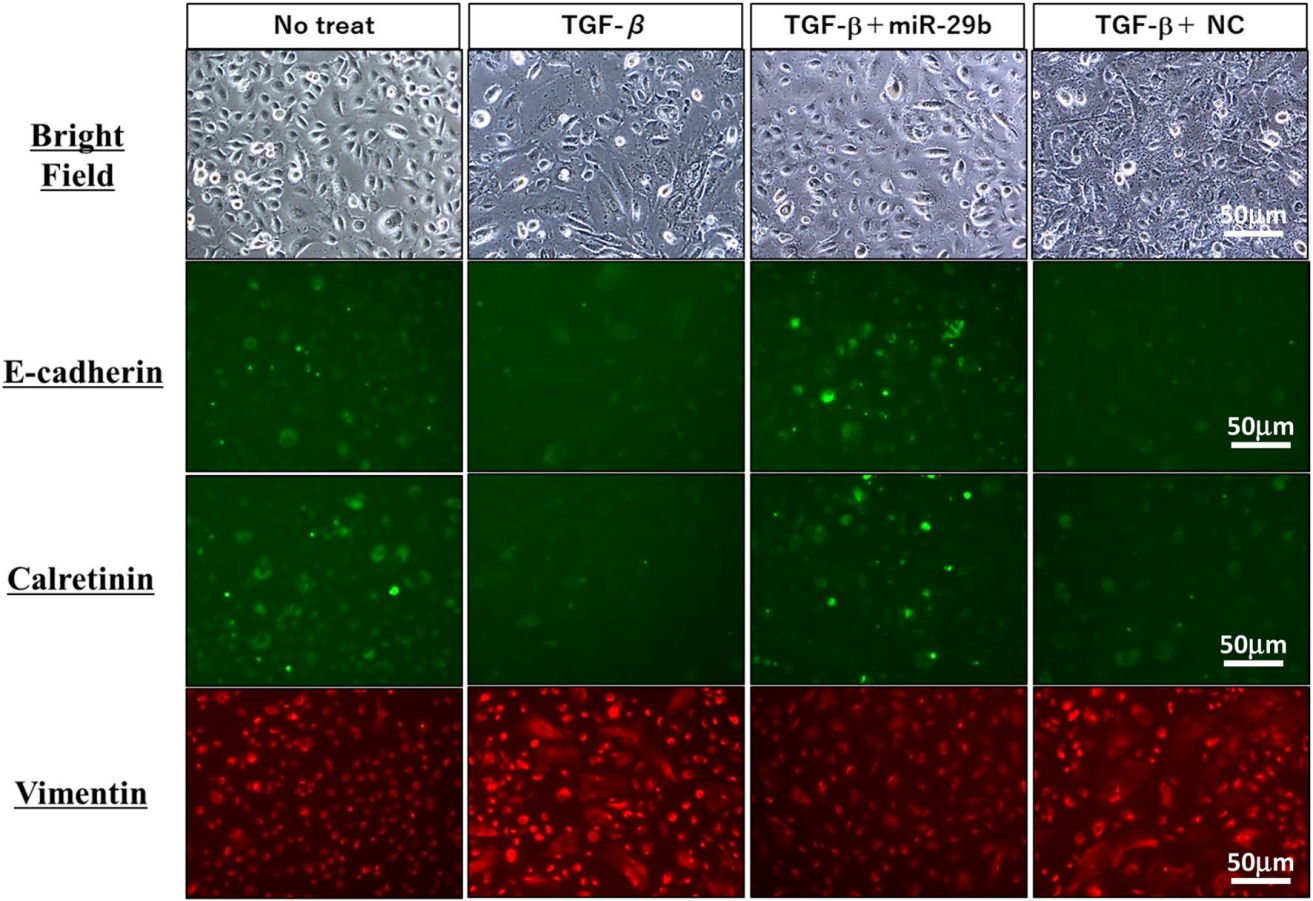
Phenotypic changes of human peritoneal mesothelial cells (HPMC) by stimulation with TGF-β1. HPMC were cultured with 10 ng/ml TGF-β1 for 48 h. In some wells, HPMC were transfected with miR-29b-3p mimic or negative control (NC) with lipofectamine RNAiMAX at a final concentration of 50 nM, and cultured with TGF-β1 for 48 h at 37 °C. Morphological changes by light microscopy and expression of E-cadherin, calretinin and vimentin were observed with a fluorescein microscopy, BZ-X710 (Keyense, Osaka, JAPAN). Magnification: ×400. All the figures were generated using BZ-H3A software (Keyense, Osaka, JAPAN) (https://www.keyence.com/products/microscope/fluorescence-microscope/bz-x700/models/bz-h3ae/).

After 48 h-culture with TGF-β1, HPMCs changed their morphology from round to spindle shape (Fig. 2). Stimulation with TGF-β1 significantly downregulated the expression of E-cadherin and calretinin, while markedly enhancing vimentin expression. However, lipofection of miR-29b mimics, but not with NC significantly suppressed the morphological changes with reduced expression of vimentin and restored the expression of E-cadherin and calretinin induced by TGF-β1 (Fig. 2). The changes of E cadherin and vimentin expression were confirmed in western blotting (Supplementary Fig. 2A).

### Transfection of miR-29b mimic suppresses the proliferation and migration of HPMCs

**Figure 3:**
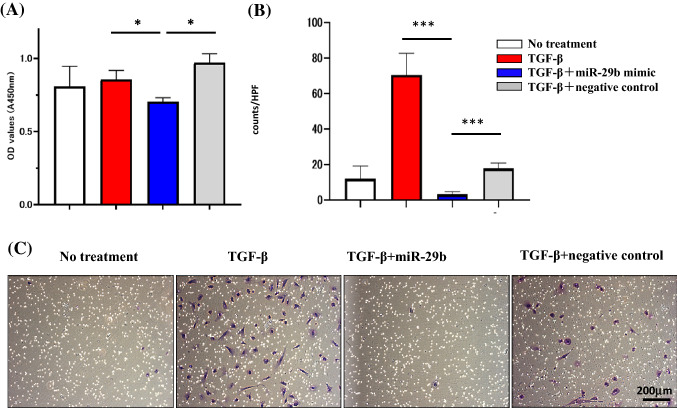
MiR-29b inhibits proliferation and migration of human peritoneal mesothelial cells (HPMC). (**A**) HPMC were cultured as described in the legend of Fig. 2 and proliferation for 3 h was determined by the MTT assay. Data show mean ± standard deviation in 1 of the 3 different experiments. (**B**) HPMC were re-suspended in DMEM and seeded on 8-µm pore membrane in 24-well plate and 10% FBS was added to the lower chamber as a chemoattractant and the number of cells that migrated to the lower surface of culture insets were counted. Data show mean ± standard deviation in 1 of the 3 different experiments. Photos were taken with an inverted microscope IX73 (Olympus, Tokyo, JAPAN). *p < 0.05, ***p < 0.001.

The proliferation of HPMC was not significantly altered by TGF-β. However, transfection with miR-29b slightly decreased proliferation (n = 3, p < 0.05) (Fig. 3A). Although HPMC rarely migrated to the lower surface of culture inserts, they did migrate to 10% FCS after stimulation with 10 ng/ml TGF-β1 (12.0 ± 7.2 vs 70.5 ± 12.2 counts/HPF, n = 3, p < 0.001). When pretreated with miR29b mimic, the number of migrated cells was greatly reduced compared with negative control miRNA (3.4 ± 1.3 vs 17.9 ± 3.1 counts/HPF, n = 3, p < 0.001) (Fig. 3B,C).

### Transfection of miR-29b mimic in HPMC suppresses tumor cell adhesion mediated by the RGD/β1 integrin cascade

**Figure 4:**
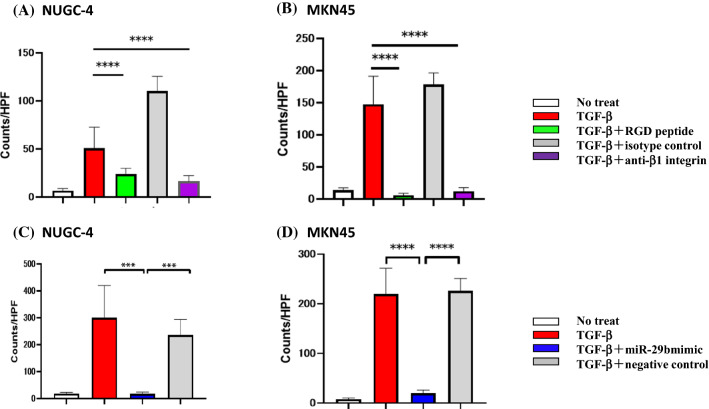
Adhesion of gastric cancer cells on human peritoneal mesothelial cells (HPMC). HPMC were cultured with TGF-β as described in the legend of Fig. 2 and then fluorescein labelled NUGC-4 and MKN45 were added, incubated for 15 min on the mesothelial cell (MC) monolayer. After gentle washing with warmed media for 3 times, the number of NUGC-4 cells remaining attached were counted under a fluorescent microscope. In (**A**) and (**B**), MC monolayer was incubated with RGD peptide (40 μM), anti-β1 integrin mAb or isotype control mouse IgG (20 μg/ml) before the addition of tumor cells. In (**C**) and (**D**), MC monolayer was pretreated with miR-29b mimic or negative control as described in the legend of Fig. 2. Data show mean ± standard deviation in 1 of the 2 different experiments ***p < 0.001, ****p < 0.0001.

In adhesion experiments, a few NUGC-4 and MKN45 attached to the HPMC monolayer. However, after stimulation of HPMC with TGF-β1, number of NUGC-4 and MKN45 attached to the HPMC markedly increased (NUGC-4, 6.7 ± 2.3 vs 50.7 ± 22.0 counts/HPF, n = 5, p < 0.001; MKN45; 13.7 ± 3.7 vs 147.9 ± 43.4 counts/HPF, n = 5, p < 0.0001) (Fig. 4A,B). However, enhanced adhesion of NUGC-4 and MKN45 was significantly reduced by the presence of RGD peptide (NUGC-4; 23.9 ± 5.9 counts/HPF, n = 5, p < 0.0001, MKN45; 6.2 ± 2.7 counts/HPF, n = 5, p < 0.0001) or anti-β1 integrin mAb (NUGC-4; 16.3 ± 6.1 counts/HPF, n = 5, p < 0.0001; MKN45; 12.3 ± 5.4 counts/HPF, n = 5, p < 0.0001) (Fig. 4A,B).

When HPMC were transfected with miR-29b, but not with negative control, the enhancing effects of TGF-β1 on the adhesion of NUGC-4 and MKN45 were totally abrogated (NUGC-4; 18.0 ± 5.2 vs 300.0 ± 119.8 counts/HPF, n = 5, p < 0.001; MKN45, 7.5 ± 2.4 vs 219.9 ± 51.7 counts/HPF, n = 5, p < 0.0001) (Fig. 4C,D).

### Transfection of miR-29b mimic to HPMC suppresses the expression of fibronectin (FN)

**Figure 5:**
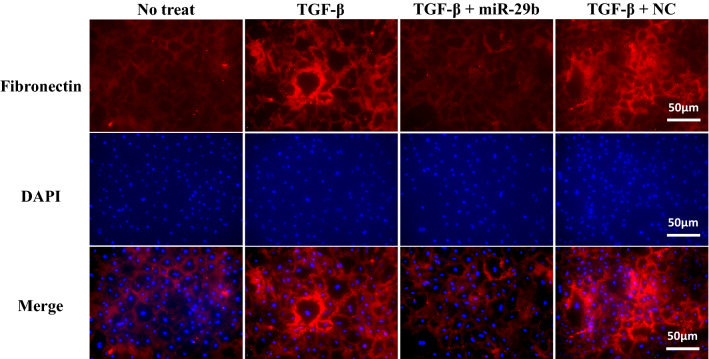
Expression of fibronectin (FN) on human peritoneal mesothelial cells (HPMC). HPMC were transfected with miR-29b-3p mimic or negative control (NC) or treated with lipofectaminealone (MOC) as described in the legend of Fig. 2, immunostained with DAPI and anti-FN mAb and surface expression of FN examined with fluorescein microscopy. Red; FN; Blue, nuclear deoxyribonucleic acid (DNA). ipofectamine RNAiMAX Magnification: ×400. All the figures were generated using BZ-H3A software (Keyense, Osaka, JAPAN) as shown in the legend of Fig. 2.

Since adhesion data suggest a contribution of fibronectin (FN) in this adhesion cascade, we next examined the effect on the expression of FN on HPMC. FN was faintly detected on the surface of resting HPMC by immunohistochemistry analysis. However, after stimulation with TGF-β1, HPMC strongly expressed FN (Fig. 5). When miR-29b mimics were transfected with TGF-β1 stimulation, the expression of FN was not increased (Fig. 5). The change of FN expression was confirmed in western blotting (Supplementary Fig. 2B).

### Transfection of miR-29b mimic to HPMC suppresses the transmigration of NUGC-4 through HPMC

**Figure 6:**
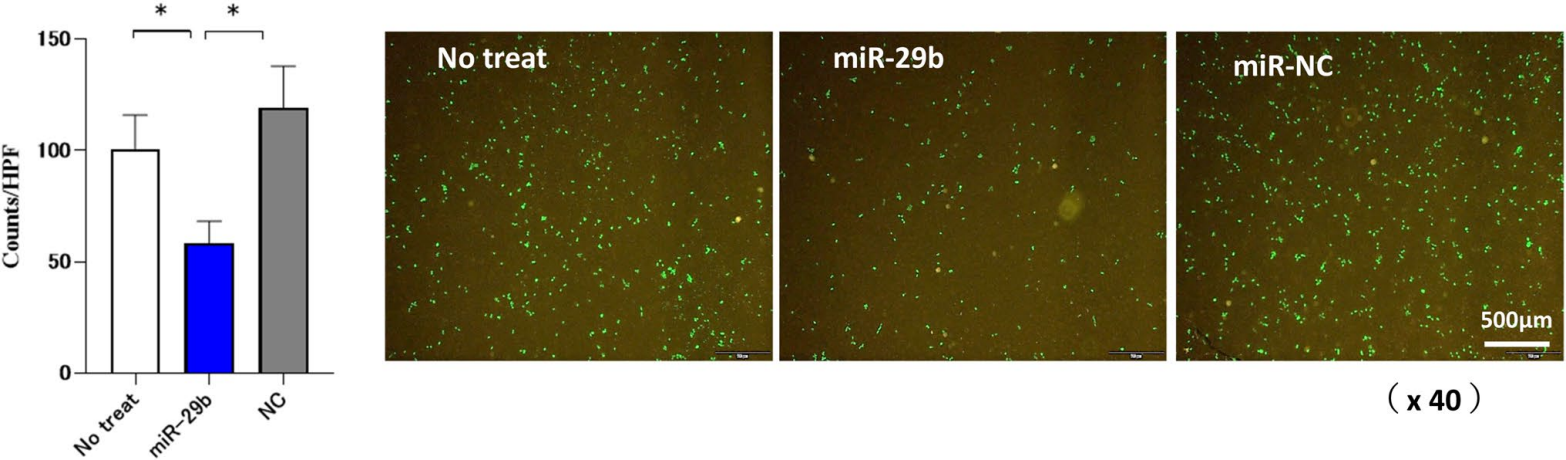
MiR-29b inhibits transmigration of NUGC through HPMC monolayer. The HPMC were seeded on the culture inserts with 8-µm pore membrane in 24-well plate and miR-29b were transfected with miR-29b-3p mimic or negative control (NC). After washing the media, GFP-transfected NUGC-4 suspended with DMEM (1 × 105/100 μl) were added on the top chamber and 20% FBS was added as a chemoattractant in the lower chamber, After the co incubation for another 72 h, NUGC-4 cells migrating to the lower surface of chamber were counted under fluorescein microscopy. Data show mean ± standard deviation in a representative experiment. Figures were generated using BZ-H3A software (Keyense, Osaka, JAPAN) as shown in the legend of Fig. 2. *p < 0.05.

Next, we examined the transmigration of GFP-labelled NUGC-4 through HPMC monolayer with double chamber system. When HPMC was cultured type 1 collagen gel and NUGC-4 on it, we observed that HPMC preceded NUGC in invading to deep area (Supplementary Fig. 3). As shown in Fig. 6, the pretreatment of HPMC with miR-29b mimics significantly reduced the transmigration of NUGC-4 (100.8 ± 15.1 counts/HPF vs 58.5 ± 9.8 counts/HPF, p < 0.05).

### MiR-29b mimic partially suppresses peritoneal metastases in vivo

**Figure 7:**
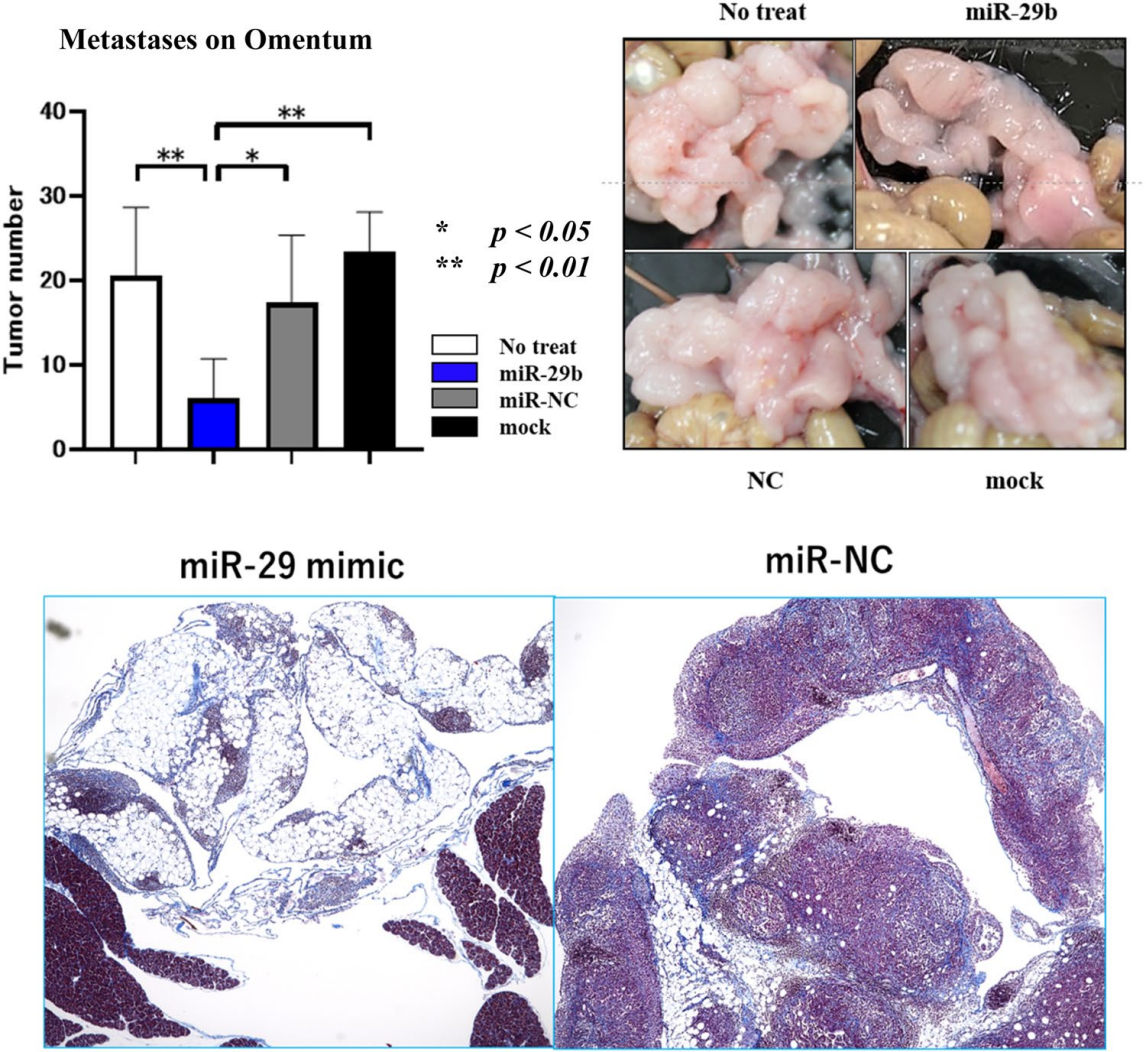
Mouse gastric cancer cell (YTN16P2, 1 × 106) were injected into the abdomen of syngenic C57/BL6 mouse and metastases to omental tissue was examined. miR-29b or negative control (miR-NC) (2 μg) were mixed with atelocollagen (80 μl) and injected at day 3, 6, 9, 12. The mice were sacrificed at day 14 and metastatic nodules on omental tissue were counted (upper). Histological appearance of omentum of miR-29b or miR-NC treated mice with H.E staining (lower).

Finally, we examined the effects of local transfer of miR-29b mimics on PM in vivo using syngenic mouse model. When miR-29b mimics were mixed with atelocollagen and intraperitoneally injected, metastatic nodules of YTN16P2 on mesentery did not show marked difference (data not shown). However, the volumes of metastases on omental tissue were significantly decreased as compared with negative control (miR-NC) (Fig. 7).

## Discussion

MC cover the inner surface of the peritoneal cavity and secrete large amounts of lubricant that facilitates intracoelomic movement and actively contributes to fluid transport, coagulation, fibrinolysis and local immunity^25^. MC are initially considered to be a mechanical barrier to tumor metastases in the peritoneal space^26,27^. However, recent studies have suggested that MC activated by TGF-β acquire a mesenchymal-like phenotype through the MMT which mediate attachment and enhance the migration of disseminated tumor cells to the sub-peritoneal space like cancer associated fibroblasts (CAFs)^28–30^.

In this study, we confirmed that HPMC acquire the phenotype of CAF with enhanced proliferative and migratory properties by TGF-β1 stimulation. However, all of the observed phenomena were largely abrogated by the transfection of miR-29b. Extensive studies have already shown that the miR-29 family regulates multiple oncogenic processes, including epigenetics, proteostasis, metabolism, proliferation, apoptosis, metastasis, fibrosis, angiogenesis, and immunomodulation^15,17^. In particular, miR-29 strongly suppresses the epithelial mesenchymal transition (EMT) of tumor cells^19,31^ through targeting the expression of various genes including catenin, β1-integrin and matrix metalloproteinases (MMPs)^15,17^. Given that these EMT-related genes are largely shared with MC, the results of this study indicate that TGF-β1 induces MMT mainly through the downregulation of miR-29b.

Treatment with TGF-β1 markedly upregulated FN expression on an HPMC monolayer and induced robust adhesion of gastric cancer cells which was inhibited by an antibody to β1 integrin and RGD peptide. This is consistent with the results in previous studies showing that FN-β1 integrin is an important adhesion pathway between MC and tumor cell^30,32^. However, the enhanced adhesion by TGF-β1 was also abrogated by transfection of miR-29b. The miR-29 family has been shown to suppress the synthesis of extracellular matrix proteins such as collagens, laminin and fibrillin in embryonic fibroblasts^33^, trabecular meshwork cells^34^ and hepatic stellate cells^35^. Zhu et al. showed that miR-29b downregulates FN production via Hsp47 in breast cancer cells^36^. The results of the present study are consistent with those results and suggest that transduction of miR-29b in MC effectively inhibits the attachment and subsequent migration of disseminated tumor cells on MC which is the initial step in the development of PM.

In previous studies, we found that the expression of exosomal miR-29b in peritoneal fluid was markedly reduced in patients who developed peritoneal recurrence after curative resection of gastric cancer^37^ as well as patients with PM^38^. Generally, expression of miR-29 in tumor cells decreased mainly via hypermethylation of the CpG areas of the miR-29 promoter lesion^39,40^, and the diminished expression of miR-29 is believed to promote tumor progression^16,17^. However, since the number of disseminated tumor cells is thought to be low during the initial step of the development of PM, it is unlikely that reduced miR-29b in peritoneal fluids is dependent on tumor cells.

In this study, we found that resting HPMC produce substantial amount of miR-29b which was markedly suppressed by the addition of TGF-β1. This is consistent with previous studies showing that the TGF-β-SMAD signal suppresses miR-29 expression^41,42^ and considerable levels of TGF-β could be detected in malignant effusions^43,44^. These facts suggest that reduced production of miR-29b in MC downregulates the amount of miR-29b in the abdominal cavity which is a prerequisite for the development of PM. Taken together, this suggests that replacement of miR-29b in peritoneal space may be effective to prevent PM partly through its effects on MC. In fact, intraperitoneal administration of miR-29b mimics using atelocollagen partially suppressed metastases, at least, in omentum. In vivo study using appropriate delivery methods is warranted.

## Methods

### Reagents

The oligonucleotides of miR-29b-3p mimics and negative control miRNA were synthesized by Thermo Fisher Scientific (Waltham, MA). The sequence of the oligonucleotides used for miR-29b-3pmimics.

5′-UAGCACCAUUUGAAAUCAGUGUU-3.

Lipofectamine RNAiMAX was purchased from Invitrogen (Carlsbad, CA). Rabbit mAbs to E-cadherin and calretinin, were purchased from Abcam (Cambridge, UK), Cell Signaling Technology (Danvers, MA). Recombinant- TGF-β1 was purchased from R&D systems (Minneapolis, MN). Rabbit anti-vimentin mAb and anti-rabbit Ig conjugated with AlexaFluor 488 or AlexaFluor 595® were from Invitrogen. DAPI was obtained from Dojindo (Kumamoto, Japan). Anti-integrin β1 mAb and RGD peptide were purchased from Cayman Chemical Co. (Ann Arbor, MI).

All methods were carried out in accordance with guidelines and regulations of the Declaration of Helsinki and all experimental protocols were approved by the Institutional Review Boards of Jichi Medical University (Approval number: RIN-A-19-161).

### Cell culture

Human gastric cancer cells, NUGC-4, MKN45 were obtained from Riken (Tsukuba JAPAN), and a bone marrow derived mesenchymal stem cell line, UE6E7T-12, was obtained from the Japan Health Science Foundation (Tokyo, Japan). The cells were cultured in Dulbecco’s Modified Eagle Medium (DMEM) supplemented with 10% fetal bovine serum (FBS; Sigma, St. Louis, MO), 100 U/mL penicillin, and 100 mg/mL streptomycin (Life Technologies, Grand Island, NY) at 37 °C in a 5% CO_2_ cell culture incubator.

### Isolation and culture of human peritoneal mesothelial cells (HPMC)

HPMC were isolated from 2 to 4 cm^3^ samples of omentum collected from consenting patients undergoing sleeve gastrectomy. Omental samples were momentarily placed in a half TrypLE Express (Thermo Fisher Scientific, Waltham, MA) with pure PBS, and incubated in a thermostatic tank at 37 °C for 2 h. The supernatants were collected after filtration by 100 µm-pore nylon mesh, were centrifuged at 1500 rpm and 4 °C for 5 min. Explants were seeded into collagen coated 10 cm^2^ tissue culture dishes (Falcon, Becton Dickinson, Oxford, UK) and cultured in DMEM with 20% FBS, 100 U/mL penicillin, and 100 mg/mL streptomycin at 37 °C in 5% CO_2_ cell culture incubator (Riera, McCulloch et al. 2006). Informed consent was obtained from all study participants.

### Sample collection and miRNA purification and digital polymerase reaction (PCR)

Cell line miRNAs were isolated from cell pellets using the MiRNeasy kit (Qiagen, Hilden, Germany), according to the manufactures’ instructions. cDNA was synthesized starting from 100 μg of extracted RNA using the TaqMan miRNA Reverse Transcription Kit and miRNA-specific stem-loop primers (Applied BioSystems, Foster City, CA) following manufacturer’s instructions. Total miR-29b-3p cell levels were then quantified using the digital (dd) PCR system (Bio-Rad Laboratories, Hercules, CA). Briefly, 10 μg of synthesized cDNA were added to a 20 μl PCR reaction mixture containing 10 μl of digital PCR™ supermix (Bio-Rad Laboratories), 1 μl of TaqMan primer/probe mix (Applied BioSystems) and RNase-free H2O. Droplets were generated by loading the mixture onto a plastic cartridge with 70 μl of QX100 Droplet Generation oil (Bio-Rad Laboratories). Cartridges were then placed into the QX200 Droplet Generator (Bio-Rad Laboratories). The droplets generated from each sample were transferred to a 96-well PCR plate (Eppendorf, Hamburg, Germany), and PCR amplification was carried out on the C1000 Touch Thermal Cycler (Bio-Rad Laboratories), according to the manufacturer’s protocol. The plate was then loaded on the QX200 Droplet Reader (Bio-Rad Laboratories) and read automatically. The fraction of PCR-positive droplets was quantified assuming a Poisson distribution. QuantaSoft software was used to obtain the concentration results in number of copies per microliter for each sample.

### Introduction of MMT and transfection of miR-29b-3p

HPMCs were seeded in 6-well plates at 50–60% confluence and induced with 10 ng/ml of TGF-β1. Then, the cells were transfected with miR-29b-3p mimic or negative controls with lipofectamine RNAiMAX in HPMC at a final concentration of 50 nM, and cultured for 48 h at 37 °C, according to the manufacturer’s instructions and then used for the following experiments.

### Immunofluorescence

HPMC (5 × 10^4^) were plated in 24-well collagen coated plates, incubated with 10 ng/ml of TGF-β1 and then transfected miR-29b or negative controls for 48 h. Cells were washed with PBS, fixed in 4% paraformaldehyde for 10 min at 37 °C and then permeabilized with 0.5% Tween-20 in PBS for 20 min. Subsequently, cells were blocked for 1 h with 3% BSA in PBS at room temperature. Cells were then incubated for 1 h at room temperature with mAbs to E-cadherin (1:200), calretinin (1:500), vimentin (1:1000), and fibronectin (FN) (1:150). Then, cells were washed 3 times with PBS and incubated for 30 min at room temperature with the appropriate fluorescence conjugated secondary anti-rabbit antibodies conjugated with AlexaFluor 488 or AlexaFluor 595^®^ (1:2000). Lastly, the nucleus was counterstained with DAPI (1:1000) for 5 min. Glass coverslips were placed on slides and the preparations were visualized under a fluorescence microscope BZ-X710 (Keyense, Osaka, JAPAN) and the figures were generated using BZ-H3A software (Keyense, Osaka, JAPAN) (https://www.keyence.com/products/microscope/fluorescence-microscope/bz-x700/models/bz-h3ae/).

### Cell proliferation

HPMCs (1.0 × 10^4^ cells) transfected with miR-29b or negative controls were cultured with or without TGF-β1 in 96-well culture plate for 24 h and incubated with MTS (DOJINDO, Kumamoto, Japan) diluted in normal culture media at 37 °C for an additional 3 h. Proliferation rates were determined and quantification was performed on a microtiter plate reader (Spectra Rainbow; Tecan) according to the manufacturer’s protocol.

### Cell migration

HPMCs (5.0 × 10^5^ cells) transfected with miR-29b or negative controls were cultured with or without TGF-β1 for 48 h. Cells were re-suspended in DMEM and seeded on an 8-µm pore membrane in 24-well plate and 10% FBS was added to the lower chamber as a chemoattractant. After 24 h, non-migrated cells were gently removed with a cotton swab. Migrated cells on the lower surface of the culture insets were stained with Dif-Quick (Sysmex, Kobe, Japan) and counted. In the analysis of migration of tumor cells, GFP-labelled NUGC-4 were placed on the HPMC monolayer and incubated for 72 h and cells migrating to the lower surface were counted under fluorescein microscopy.

### Cell adhesion assay

HPMCs (5.0 × 10^4^ cells) transfected with miR-29b or negative controls were cultured with or without TGF-β1 in 24-well culture plates. Fluorescein labelled NUGC-4 (1.0 × 10^4^ cells/well) were then added, incubated for 15 min for all cells to attach to the MC monolayer. After gentle washing with warmed media 3 times, the number of NUGC cells remaining attached were counted under a fluorescent microscope.

### In vivo experiment

YTN16 established in p53 heterozygous knockout C57BL/6 mice by oral intake of *N*-Methyl-*N*-nitrosourea (MNU) was a generous gift from Dr S. Nomura (Tokyo University)^45^. YTN16P2 was a highly metastatic a subline of YTN16 by in vivo selection method. The YTN16P2 (1 × 10^6^) were intraperitoneally (IP) transferred in C57BL/6 mice and treated with IP local injection miR-29b or negative control (miR-NC) (2 μg) were mixed with atelocollagen (80 μl) at day 3, 6, 9, 12. The mice were sacrificed at day 14 and metastatic nodules on omental tissue or mesentery were counted. All procedures were approved by the Animal Care Committee of Jichi Medical University (No 20043-01) and performed in accordance with ARRIVE guidelines.

### Statistical analysis

Data were represented as mean ± standard deviation. The significance of the differences between groups was assessed with a one-way ANOVA using GraphPad Prism8. Differences were considered significant when P < 0.05.

## Supplementary Information

Supplementary Figures.
