# Supplementary material for: MiR-29b may suppresses peritoneal metastases through inhibition of the mesothelial–mesenchymal transition (MMT) of human peritoneal mesothelial cells
Source: Sci Rep. 2022 Jan 7;12:205. doi: 10.1038/s41598-021-04065-2 (PMC8742040; doi:10.1038/s41598-021-04065-2)
Supplement: Supplementary file 1 — Supplementary Figures. [file 41598_2021_4065_MOESM1_ESM.docx]

Supplementary Figure 1


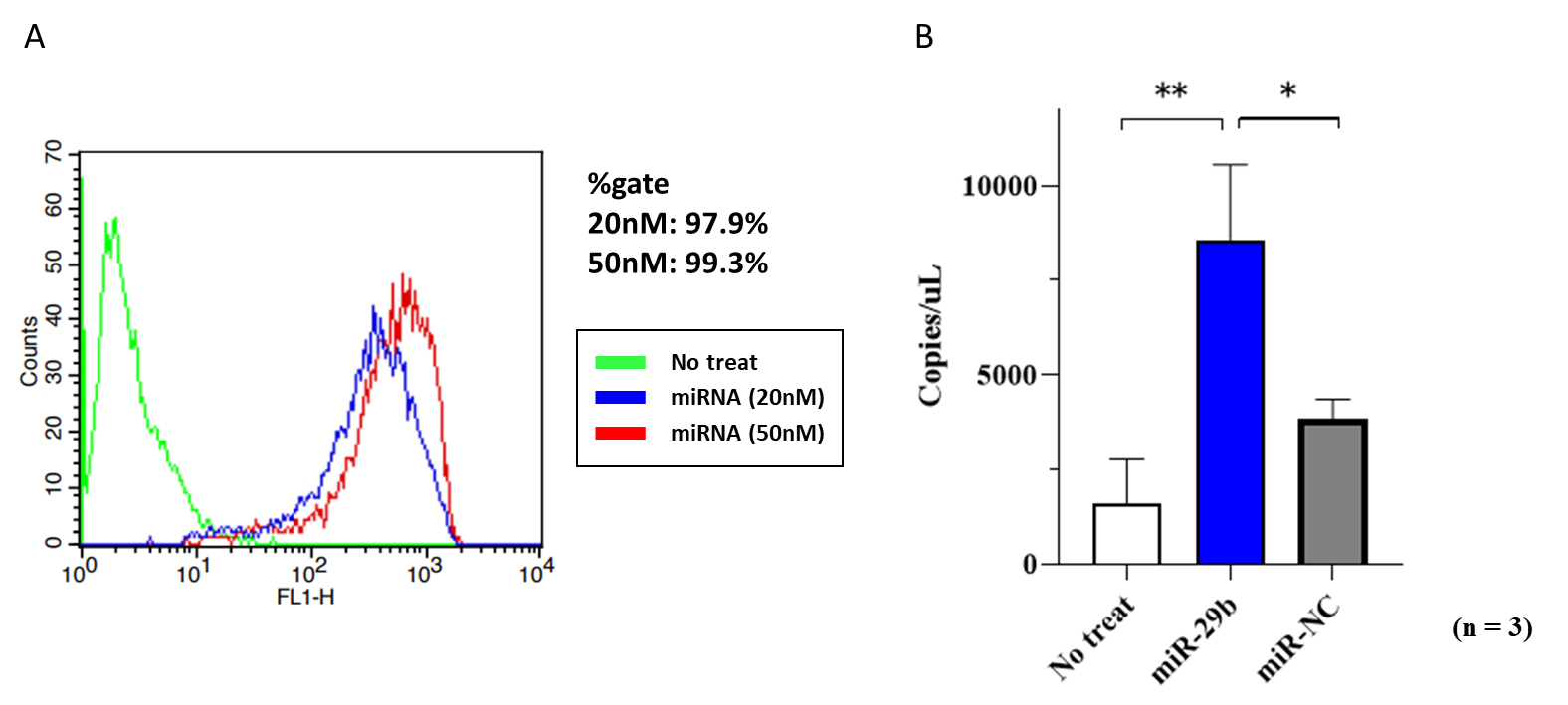


Human peritoneal mesothelial cell (HPMC) efficiently captures miRNA introduced by the lipofection method.

A: HPMC were transfected with the siRNA-selective fluorescent probe or without with Lipofectamine RNAiMAX at a final concentration of 20nM or 50nM, and cultured for 48 hr at 37°C. The percentage of fluorescence positive cell was examined by FACS. B: HPMC were transfected with miR-29b-3p mimic or negative control miR (NC) with Lipofectamine RNAiMAX at a final concentration of 50nM, and cultured for 48 hr at 37°C. Total miR-29b was quantified using the digital PCR system. *; p < 0.05, **; p < 0.01.

Supplementary Figure 2


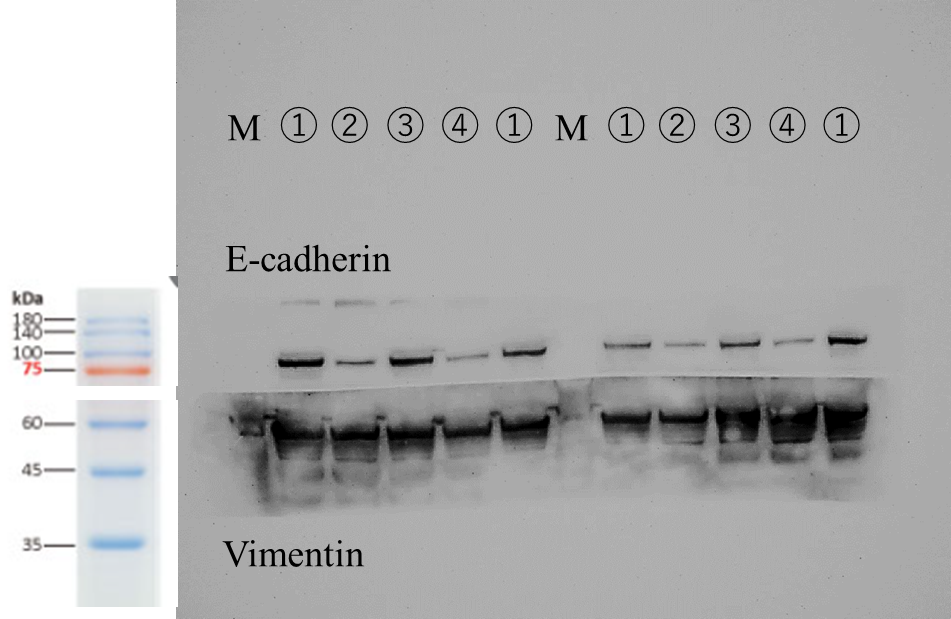


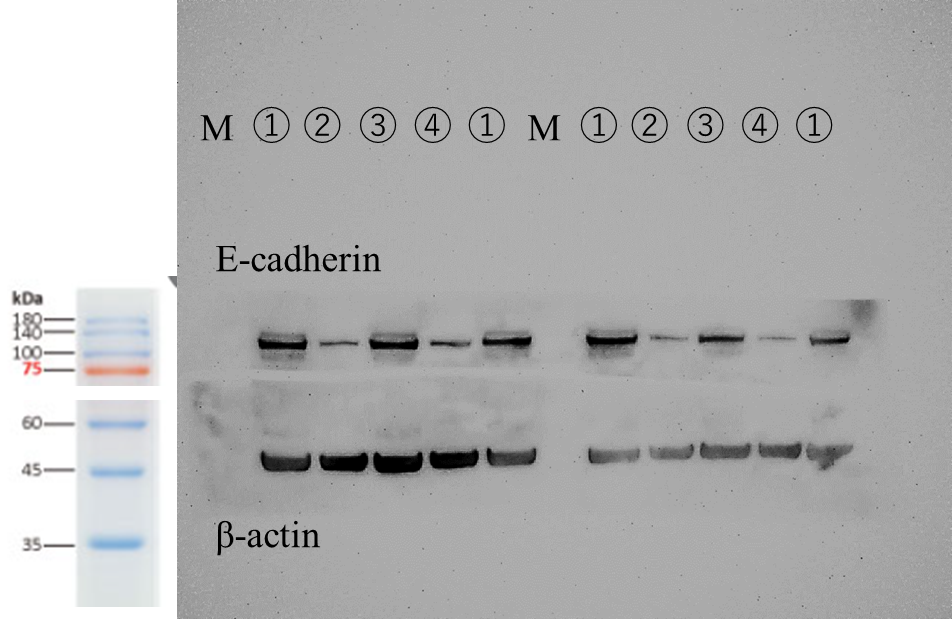


① :TGF-β1 (-) ② :TGF-β1 (+) ③ :TGF-β1+miR-29b ④ :TGF-β1+NC

M :Spectra Multicolor High Range Protein Ladder (Thermo scientific)


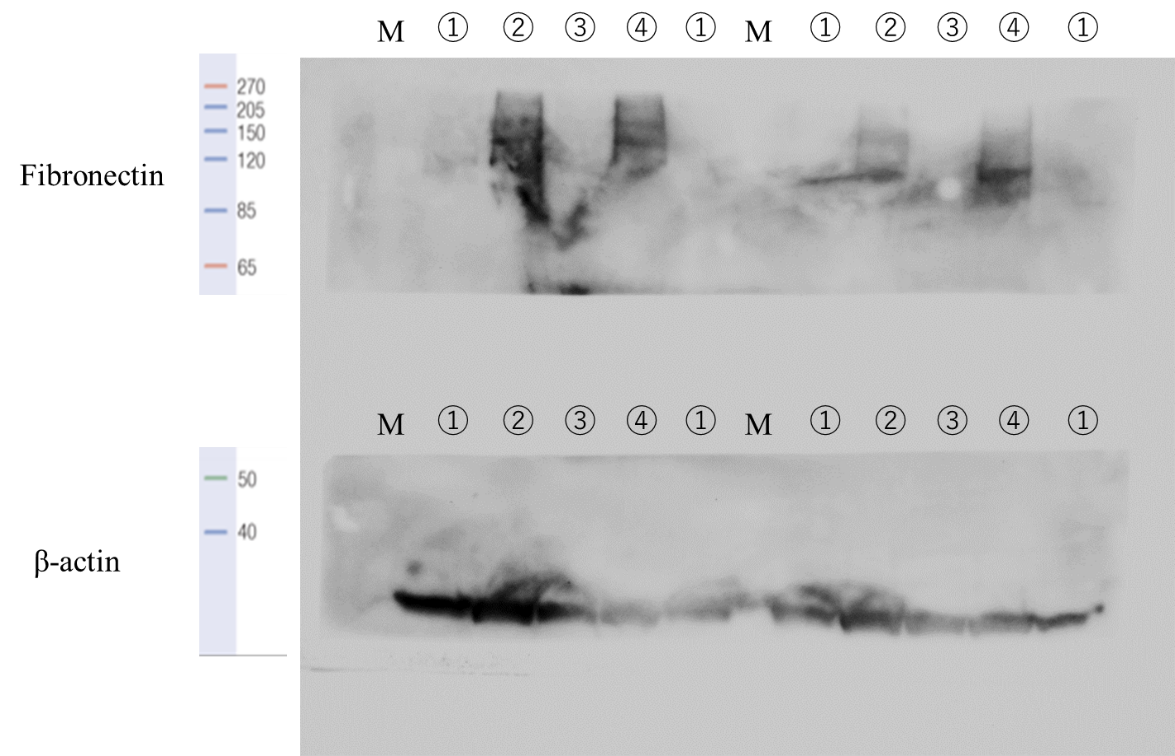


① :TGF-β1 (-) ② :TGF-β1 (+) ③ :TGF-β1+miR-29b ④ :TGF-β1+NC

M :Spectra Multicolor High Range Protein Ladder (Thermo scientific)

The protein levels of human peritoneal mesothelial cell (HPMC) were measured by western blotting. HPMC were transfection of miR-29b or negative control (NC) and cultured with or without 10ng/ml TGF-β1 for 48 hours. Total proteins extracted by protein lysis solution were appended to the loading buffer, boiled at 95 °C for 10 min (40 µg/well), and isolated with 10% sodium dodecyl sulfate polyacrylamide gel electropheresis, and transferred to PVDF membranes. Subsequently, the membrane was blocked for Blocking One (Nakarai Tesc, Kyoto), and primary antibodies for E-cadherin (1:1000, Cell Signaling Technology), Vimentin (1:500, Invitrogen, Carlsbad, CA), Fibronectin 1 (FN1) (1:1000, Abcam, Cambridge, UK) or β-actin (1:1000, Cell Signaling Technology, Danvers, MA, USA) were added to the membrane. After cultured overnight in 4 °C , HPR-conjugated secondary antibodies (1:50000, Cytiva, Tokyo, Japan) were added. After incubated for 1 h, the image was developed by chemiluminescence reagent. β-actin was served as an internal control. Bio-rad Gel Doc EZ imager (Bio-Rad laboratories, CA, USA) was utilized to detect the band. The same samples were loaded in different gels at the same time and the gels were horizontally cut to hybridize with different antibodies.

Supplementary Figure 3


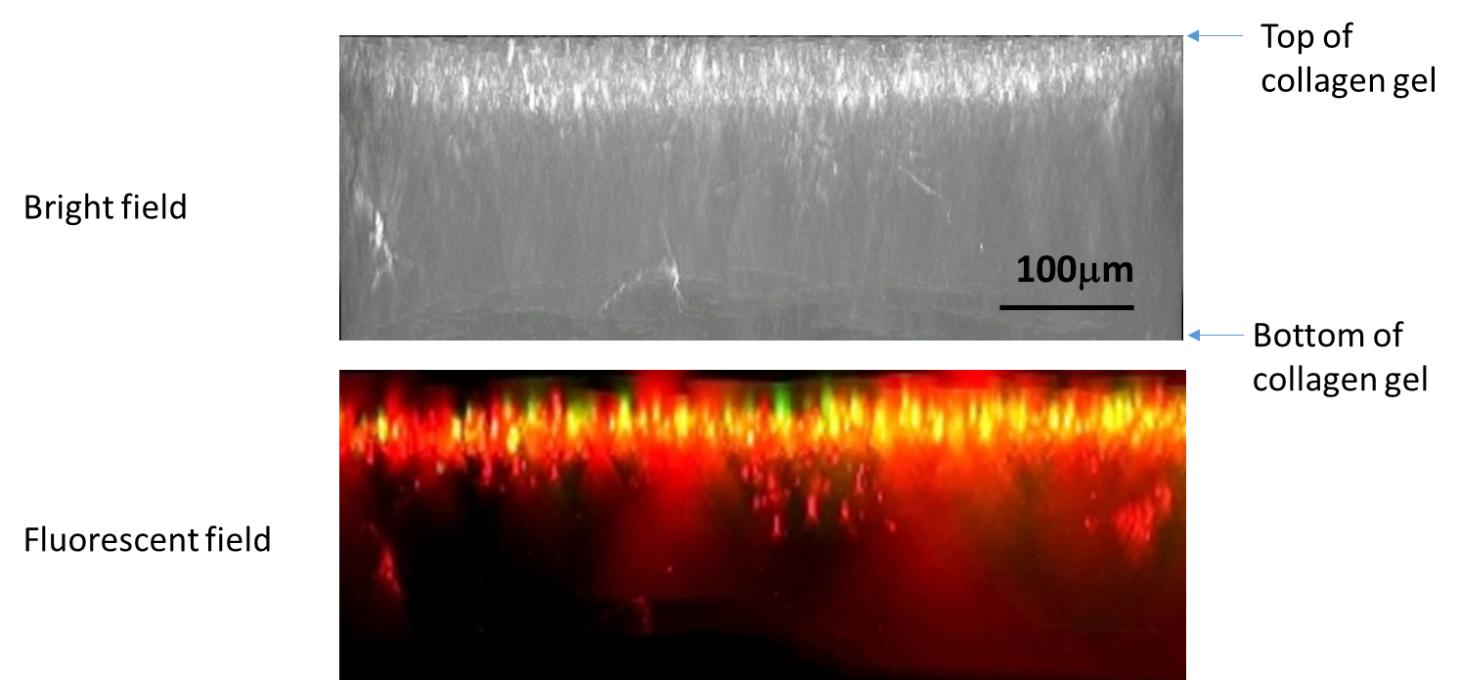


HPMC stained red by PKH26 were on Type 1 collagen gel constructed in 6 well culture inserts. After reaching confluent, GFP-labelled NUGC-4 were added on it. Invasion pattern to collagen gel to 100% FCS in lower chamber was examined at 48 hour after co-culture. Images were taken every 1mm from the top layer to the bottom layer of collagen gel with z-stack method under a fluorescence microscope, BZ-X710 (Keyense, Osaka, JAPAN), and all images were reconstructed in 3 dimension using using BZ-H3A software (Keyense, Osaka, JAPAN) (https://www.keyence.com/products/microscope/fluorescence-microscope/bz-x700/models/bz-h3ae/)

Red HPMC preceded green NUGC in invading to deep area.
